# Screening of Hydrophilic Polymers Reveals Broad Activity in Protecting Phages during Cryopreservation

**DOI:** 10.1021/acs.biomac.3c01042

**Published:** 2023-12-21

**Authors:** Huba L. Marton, Apoorva Bhatt, Antonia P. Sagona, Peter Kilbride, Matthew I. Gibson

**Affiliations:** † Department of Chemistry, University of Warwick, Coventry, CV4 7AL, United Kingdom; ‡ Warwick Medical School, University of Warwick, Coventry, CV4 7AL, United Kingdom; § School of Life Sciences, University of Warwick, Coventry, CV4 7AL, United Kingdom; ∥ Asymptote, Cytiva, Chivers Way, Cambridge CB24 9BZ, United Kingdom; ⊥ School of Biosciences, University of Birmingham, Birmingham, B15 2TT, United Kingdom; # Institute of Microbiology and Infection, University of Birmingham, Birmingham, B15 2TT, United Kingdom; ○ Department of Chemistry, University of Manchester, Oxford Road, Manchester, M13 9PL, United Kingdom; □ Manchester Institute of Biotechnology, University of Manchester, 131 Princess Street, Manchester, M1 7DN, United Kingdom

## Abstract

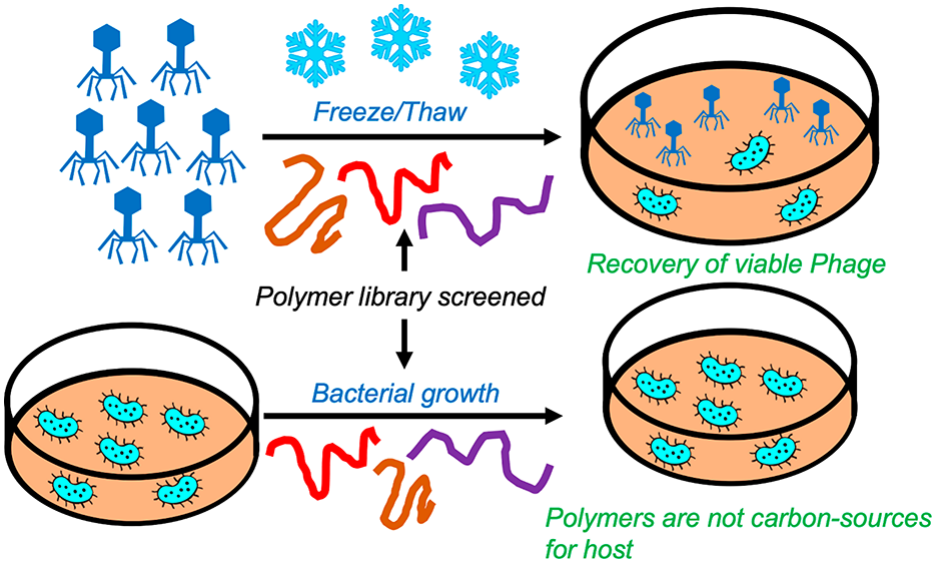

Bacteriophages have many biotechnological and therapeutic applications, but as with other biologics, cryopreservation is essential for storage and distribution. Macromolecular cryoprotectants are emerging for a range of biologics, but the chemical space for polymer-mediated phage cryopreservation has not been explored. Here we screen the cryoprotective effect of a panel of polymers against five distinct phages, showing that nearly all the tested polymers provide a benefit. Exceptions were poly(methacrylic acid) and poly(acrylic acid), which can inhibit phage-infection with bacteria, making post-thaw recovery challenging to assess. A particular benefit of a polymeric cryopreservation formulation is that the polymers do not function as carbon sources for the phage hosts (bacteria) and hence do not interfere with post-thaw measurements. This work shows that phages are amenable to protection with hydrophilic polymers and opens up new opportunities for advanced formulations for future phage therapies and to take advantage of the additional functionality brought by the polymers.

## Introduction

The use of biological therapies (e.g., cells, proteins, viruses, vaccines) to treat disease is rapidly growing, but there remain large challenges to deliver them intact and functional to a patient or for other biotechnological applications.^1−5^ Bacteriophages, also known as phages, are viruses that specifically target and infect bacteria and are recognized as the most abundant organisms on earth.^6^ Viral and bacterial host competition drives evolutionary adaptations and diversification seen in bacteria.^7,8^ For phages, the diversity is seen in size, morphology, and genomic organization.^9−11^ Generally, phages can be divided into virulent and temperate, the former carrying out a lytic replication cycle, where the phage uses the bacterial host to replicate by seizing the host’s molecular machinery before escaping the cell to find a fresh host, the latter integrating and remaining dormant in the host genome as a “prophage” and replicating with the genome in a lysogenic cycle.^12^ Bacteriophages are ubiquitous, with prominent sources being hospital effluents^13,14^ and sewage sites.^15,16^ Some applications of phage include alleviation of pathogenic bacteria in a wide range of fish and shellfish in aquacultures^17^ and food additives (approved by the Food and Drug Administration) in meat products to protect against *Listeria monocytogenes*.^18^ Another application of lytic phages is to treat bacterial infections inside the human body, known as phage therapy.^13^ One advantage of phage therapy is the large application without disruptions to the gut microbiota.^19^ A vast abundance of the phages in nature^20^ also ensures a nearly endless pipeline enabling application as “cocktails”, thereby reducing the chances of resistance developing to an individual treatment.^21−23^ Some recent clinical studies including treatment of *Staphylococcus aureus* in prosthesis infections,^24^*Mycobacterium abscessus*, *Burkholderia dolosa*, and *Achromobacter xylosoxidans* in lung transplants,^25−27^*Acinetobacter baumannii* in pneumonia,^28^ and *Klebsiella pneumoniae* in fracture-related infections.^29^ Phase II clinical trials have also been undertaken, including the European Phage Therapy Unit (PTU) between 2008 and 2010 reporting full recovery or clinical improvement in 40% of patients (157 total),^30^ treatment of leg ulcer pathogens using Intralytix phage cocktail WPP-201 (targeting *E. coli*, *S. aureus*, and *Pseudomonas aeruginosa*), which reported no side effects,^31^ and some currently ongoing clinical trials.^32^ While the above show promising results, no phage therapy has reached Phase III clinical trials (to the best of our knowledge) or been used as mainstream antibacterial treatments in the U.S.A. or EU.^33^ This can be partially attributed to discrepancy between *in vitro* and *in vivo* data, a lack of understanding of the complex relationship between bacteriophages, bacteria, and human host^34−36^ and regulatory, commercial production and translation barriers. For example, there have been safety concerns of phage production for commercial use,^37^ fears of virulence factor transfer from phage’s bacterial host to the patient^38^ and problems with commercial scale up, highlighted in multiple halting of the PhagoBurn phase I/II clinical trials.^39^

An important factor to consider when producing a commercially viable treatment is storage options and stability over time (shelf life). The storage challenge has been recently highlighted during the development of COVID-19 vaccines, with several requiring sub −20 °C temperatures and integrated cold-chain infrastructure to enable global roll-out.^3^ One reliable method for phage cryopreservation is storage inside its host,^40^ but this requires usage of chloroform and vigorous vertexing to remove the host, which comes with the concern that phages are not always purified from host endotoxins and potentially toxic purification reagents.^13,41^ While ambient-temperature phage storage is possible, the longevity of this varies from phage to phage. For example, *A. baumannii* phage vPhT2 was reported to have excellent stability in lysogeny broth, but not in SM-II.^22^ Hence, finding a suitable method for long-term storage, for both purified phages and developing phage preparations, to standardize transport, storage, and use at the bedside is important for their wider adoption. Predictable cryopreservation outcomes are essential to controlling the phage dosage, including a matching composition of thawed and frozen phages in the case of phage cocktails.

In laboratory situations, the most commonly used cryoprotectants to enable frozen storage of biologics are glycerol (for phage, bacteria, and proteins) or DMSO (for mammalian cells).^42−46^

There has been considerable interest in new cryoprotectants,^47,48^ particularly those inspired by ice-binding proteins (“antifreeze proteins”),^49−51^ and macromolecular (polymeric) cryoprotectants have also emerged with unique modes of action.^52−57^ We recently demonstrated that poly(ethylene glycol), when used at just 10 mg·mL^–1^, could effectively cryopreserve phages, matching the performance of glycerol but at approximately 10-fold lower concentrations.^58^ No link was found to polymer ice recrystallization inhibition activity, suggesting a wide range of hydrophilic polymers may be suitable for phage cryopreservation, although acidic polymers have been shown to be phage-inhibitory.^59^ There is no complete study on which synthetic polymers or their structural parameters (such as molecular weight) aid phage cryopreservation.

This work deploys a library of synthetic polymers, derived from RAFT polymerization, to explore their ability to cryopreserve a panel of phages. It is observed that essentially all hydrophilic polymers can protect the phage, with the exception of poly(acrylic acid) and poly(methacrylic acid), due to their phage-inhibiting function, which complicates testing in this present application. Phage recovery was shown in both qualitative high throughput and quantitative plaque-counting, assays. A benefit of the polymers was identified that they do not act as carbon sources for the bacterial hosts, post-thaw, and show that macromolecular cryoprotectants could be deployed to help bank, distribute, and use phage-based technologies and therapies.

## Experimental Section

### Optical Density Measurements and Corrections

For preliminary optical density measurements at 600 nm (OD_600_) of the bacterial growth curves described below, a Fisher Scientific portable cell density meter, model 40 was used.

### Biological Methods

#### Viral Enrichment–Propagation of Bacteriophages

To propagate the bacteriophage isolates, *E. coli* EV36 and *E. coli* AB1157, hosts for the K1F-GFP, K1E, K1-5 and T7, T4 phage groups, respectively, were grown overnight in lysogeny broth (LB) (Sigma-Aldrich: Lennox −10 g·L^–1^ tryptone, 5 g·L^–1^ yeast extract, 5 g·L^–1^ NaCl) at 37 °C and 130 rpm. *E. coli* AB1157 was only used for the propagation of T7 and T4 phages, not as a host for any of the assays described below. The next day, 1 mL of the overnight liquid cultures was used to inoculate 50 mL of fresh LB, separately. This newly inoculated LB was incubated at 37 °C and 130 rpm until an OD_600_ (optical density at 600 nm) of 0.3 was reached. At this point, 100 μL of bacteriophage stock (separate for each phage) was added to each corresponding flask, and the samples were incubated for an addition 4 h (until full clearance of the cloudy media). The *E. coli* EV36 and AB1157 bacterial host debris were pelleted by centrifugation at 3220 g for 10 min before passing the supernatant through a 0.2 μm pore size membrane filter. The five prepared phage stocks in the LB were stored at 4 °C.

#### Cesium Chloride Purification of Bacteriophages

For the purification step, the previously described propagation assay was scaled up to 250 mL per sample by transferring the supernatant (containing the phages) to LB media. Sodium chloride was added to each phage sample to achieve a final concentration of 1 M. After a 1 h incubation on ice, each sample was centrifuged at 3220 g, and the supernatant was filtered through at 0.2 μm pore size membrane before adding PEG 8000 to a final concentration of 10% w/v. The phage samples were cooled overnight at 4 °C, before centrifugation at 25000 g at the same temperature for 1 h. Each phage pellet was resuspended in 6–7 mL of SM buffer I and passed through a 0.2 μm pore size membrane, before undergoing concentration and purification in a CsCl gradient for 20 h at 150000 g and 4 °C. Following the centrifugation, phages were concentrated into a band. The band of each phage was syringe extracted, first dialyzed in SM buffer I and twice dialyzed in SM buffer II to replace the CsCl with NaCl gradually. Purified phage samples were stored at 4 °C and used directly for each assay described below.

#### Cryopreservation

The purified *E. coli* targeting bacteriophage samples were diluted to a final concentration and volume of 1 × 10^7^ PFU·mL^–1^ in 500 μL (phage + additive aliquots; 10 mg·mL^–1^ additive concentration). On the other hand, for the mycobacteriophages, the purified lysate was directly used for the 500 μL phage + additive aliquots. After the samples were placed in −80 °C freezers (cooling rate was not recorded), the vials were left in the freezer for 13 days. After cryopreservation, each sample was thawed to 20 °C on benchtops, followed by continued storage at 4 °C.

#### Plaque Assay–Quantification of Bacteriophages

Bacteriophage titers for all five *E. coli* targeting phages were determined via a soft agar plaque assay, using 0.7% agar top lysogeny broth agar (LBA).^60^ A 100 μL aliquot of serially diluted cryopreserved phage were used to infect an equal volume of bacteria host cell lawn (∼1 × 10^8^ CFU·mL^–1^ (colony forming units)) by incubating at room temperature for 15 min before the addition of 3 mL liquid top agar (0.7% agar) and pouring over a solid 1.5% agar LBA plate. After an overnight (24 h) incubation at 37 °C, the individually distinct zones of clearance on plates (plaques) were enumerated and quantified as PFU·mL^–1^ (plaque forming units), taking into account the serial dilution from cryopreserved aliquots. The assays were carried out in triplicate, using duplicates for each biological repeat (*n* = 6).

#### Twenty-Four Hour *E. coli* EV36 Growth Curves–High-Throughput Screening

Bacteria and phage samples were grown in a FLUOstar Omega microplate reader at 37 °C taking measurements of the optical density (OD_600_ or Abs_600_) every 5 min over a 24 h period. Final concentration of 1 × 10^6^ CFU·mL^–1^ bacteria host was added to each corresponding well of a 96-well plate and grown for 4 h at 37 °C with shaking to reach the log phase. During the log phase, the tested aliquots were added to each appropriate well of the plate including 1% v/v Chemgene surface disinfectant (a positive control) and bacteriophages with a final concentration of 1 × 10^6^ PFU·mL^–1^ with or without the polymer additives. All samples were grown shaking in lysogeny broth (LB) media in a total volume of 200 μL. Data was acquired using the MARS data analysis software (version 5.10). The growth curves were carried out in triplicate, using technical duplicates for each biological repeat (*n* = 6).

#### Minimal Media Growth Curves

*E. coli* K-12 (MG1655 cells) were individually grown to mid log phase (OD_600_ of 0.2) in LB media at 37 °C with shaking (150 rpm). To starve the cells, the bacterial cultures were pelleted by centrifugation (1800 g, 10 min, 4 °C), washed three times in PBS (10 mL, 5 mL, 5 mL), resuspended in PBS, and grown overnight at 37 °C with shaking (150 rpm). Following the nutrient starvation, cells were centrifuged (1800 g, 10 min, 4 °C), washed three times in PBS and resuspended M9 minimally to an OD_600_ of 0.2. To 180 μL of starved culture, 20 μL of PPEGMA, PMA, PNIPAM, PHEA, PEG, glycerol, HES, and PVP (dissolved in SM-II buffer; 10 mg·mL^–1^) were added to test their potentials as carbon sources, used by the bacterial hosts, mimicking the buffer conditions used for bacteriophage cryopreservation. Glycerol (20 mM final concentration) was used as the positive control. Samples were grown shaking in M9 minimal media in a total volume of 200 μL. Data was obtained using the MARS data analysis software (version 5.10). The growth curves were carried out as single biological repeats using technical duplicates (*n* = 2).

#### Minimal Media Bacterial Viability Assays

*E. coli* K-12 (MG1655 cells) was grown to mid log phase (OD_600_ of 0.2) in LB at 37 °C with shaking (150 rpm), as previously described. To starve the cells, the same procedure as described above was used. Following the nutrient starvation, cells were centrifuged (1800 g, 10 min, 4 °C), washed three times in bacteria specific PBS and resuspended in M9 minimal media to an OD_600_ of 0.2. To 180 μL of starved culture, 20 μL of PMA and PEG (dissolved in SM-II buffer; 10 mg·mL^–1^) were added to test their potentials as carbon sources, used by the bacterial hosts, mimicking the buffer conditions used for bacteriophage cryopreservation. Glycerol (20 mM final concentration) was used as a positive control and PBS as negative control. Samples were grown shaking M9 minimal media in a total volume of 200 μL. At 11 time-points across a 27 h period, 20 μL aliquots were taken from the growing cultures and spotted on 1–6 dilution segmented LB plates. Plates were incubated overnight and number of colonies counter to acquire the CFU·mL^–1^ (colony forming unit) at each tested time-point. The assay was carried out as single biological repeats using technical duplicates (*n* = 2).

#### Polymer Washing Growth Curves

Bacteria + phage samples were grown as described above at 37 °C taking measurements of the optical density every five min over a 24 h period. Final concentration of 1 × 10^6^ CFU·mL^–1^ bacteria host (*E. coli* EV36 or *E. coli* K-12) was added to each corresponding well of a 96-well plate and grown for 4 h at 37 °C with shaking to reach the log phase. For the postcryopreservation polymer wash assay, the PAA and PMA incubated phage aliquots (all 5 of them) were diluted 1:10 from 10 to 0.01 mg·mL^–1^ before adding to the log phase host. For the precryopreservation PAA dilution assay, PAA was 1:2 diluted from 10 to 2.5 mg·mL^–1^ and incubated with K1F-GFP for 24 h before adding to the log phase host. All samples were grown shaking in lysogeny broth (LB) media in a total volume of 200 μL. Data was acquired using the MARS data analysis software. The growth curve assay was carried out in biological singlets and technical triplicates (*n* = 3).

## Results and Discussion

**Figure 1:**
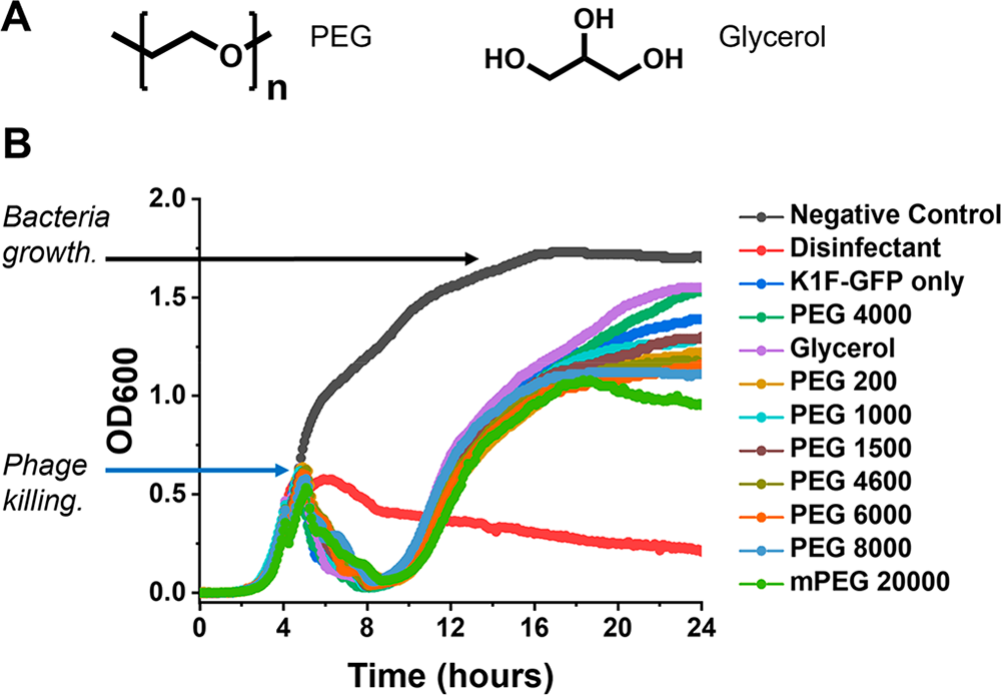
Phage cryopreservation using various molecular weights of poly(ethylene glycol). A) Chemical structures; B) Representative growth curve of PEG molecular weight screening. *E. coli* EV36 was used as the bacterial host for the K1F-GFP bacteriophage. K1F-GFP only represents the noncryopreserved phage control, LB media was used as negative and 1% Chemgene HLD4L disinfectant as positive control. All other samples were cryopreserved for 13 days. [Glycerol] and [PEG] = 10 mg.mL^–1^.

As a first test of the requirements for a polymer to cryopreserve phage, a panel of linear poly(ethylene glycol)s were tested with molecular weights from 200 to 8000 g·mol^–1^, shown in Figure 1. PEG was added to K1F-GFP phage at 10 mg.mL^–1^ and cryopreserved for 13 days at −80 °C. After this time, phages were thawed and then added to an *E. coli* EV36 host. Bacterial growth was monitored by measuring OD_600_ (optical density at 600 nm) with an increase in OD being used to indicate bacteria are healthy and growing (a negative result here) and a decrease showing bacterial killing, i.e., phage is active and has been successfully cryopreserved.^55^ In all cases, after an initial short growth period (4 h) the cryopreserved phage led to a decrease in OD, showing the phage could kill bacteria, before later recovery of the bacteria (as it is typical). This initial exploration suggested that there was no particular molecular weight fraction of PEG, which provided more protection for the phage. As a further control, poly(ethylene glycol) methyl ether (mPEG) was also tested, which gave a similar result. Therefore, it was hypothesized that any water-soluble polymer may protect the phage, and a larger library was required to explore this.

**Figure 2:**
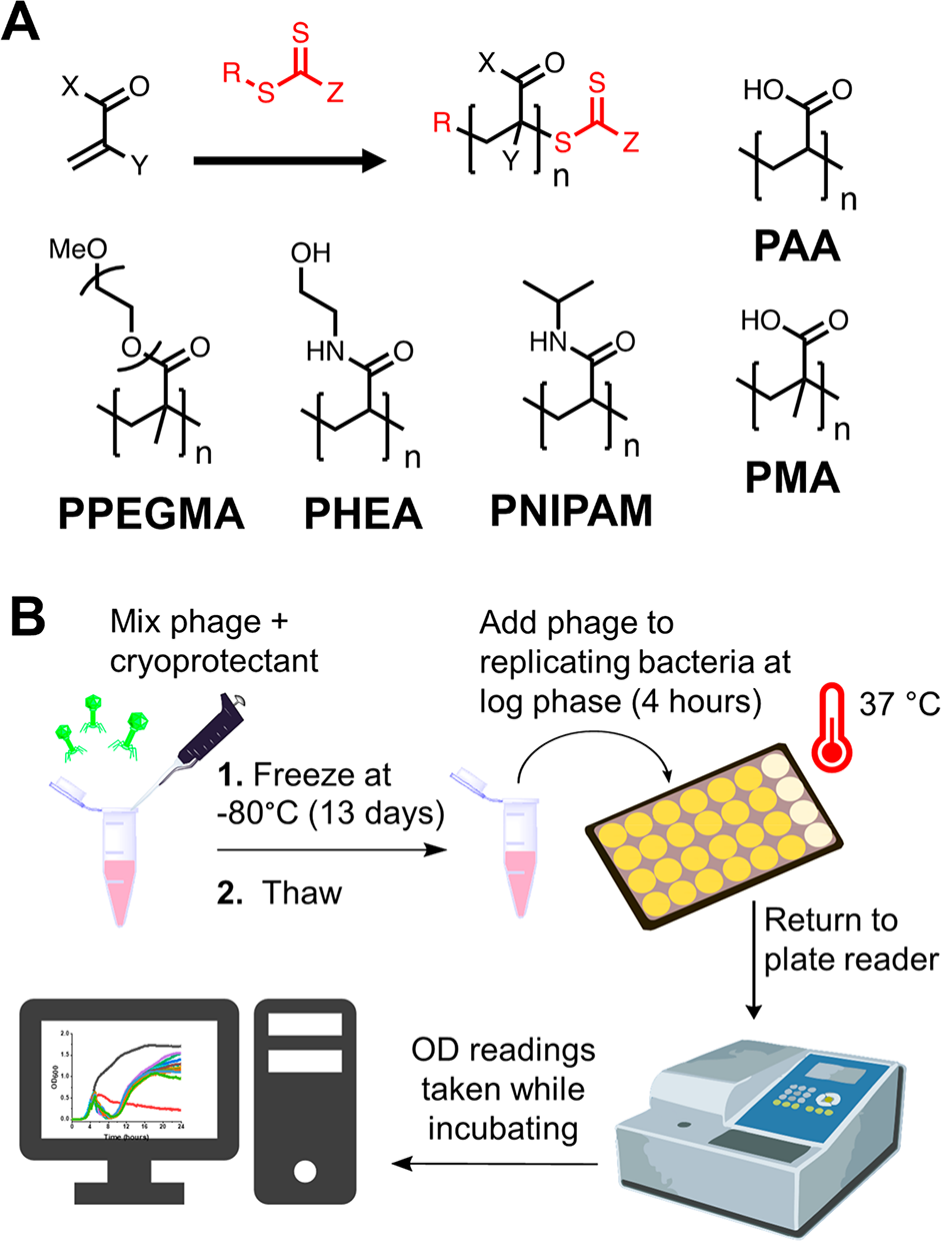
Polymer library synthesis and screening. (A) RAFT polymerization (full details in Supporting Information). (B) Schematic of freeze/thaw and high throughput screening of polymer library with the phage library (K1F-GFP, K1E, K1-5, T7, and T4 phages). Each cryopreserved sample’s total volume was 500 μL.

To enable wide chemical space to be evaluated, a panel of representative polymers were prepared by RAFT (reversible addition–fragmentation chain transfer) polymerization.^61^ It is crucial to note that cationic polymers are excluded, as these are broadly antibacterial and can act to kill the bacterial hosts of the phage and hence are not suitable.^62,63^ The panel of polymers prepared is shown in Figure 2 and Table 1. It should be noted that many of the same polymers were previously used in a study by our team to explore phage inhibiting polymers^59^ and hence identical molecular characteristics are noted. The anionic polymers (PMA and PAA) showed molecular weights from size exclusion chromatography (SEC) higher than expected from the monomer/CTA ratio. The dispersity values were low, however, suggesting either overestimation in the SEC or low initiation efficiency. This was not explored further, as the primary aim was to obtain a range of molecular weights for the phage screening (which was achieved).

**Figure 3:**
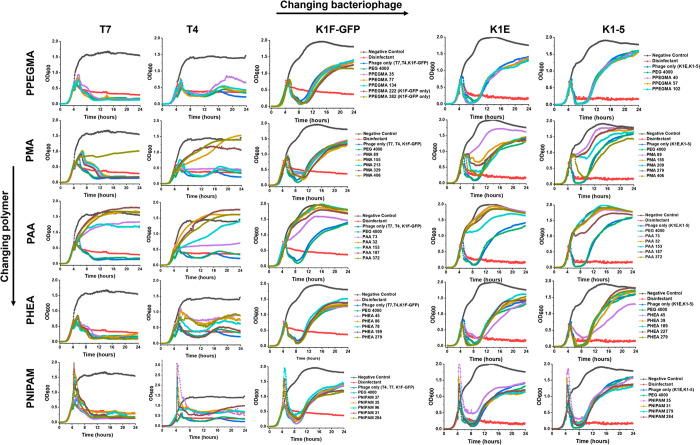
Polymer library screening for all five bacteriophages. *E. coli* EV36 was used as a host for K1F-GFP, K1E, and K1-5 phages, whereas *E. coli* K-12 (MG1655 cells) was used as a host for T7 and T4 bacteriophages, with a starting concentration of 1 × 10^6^ CFU·mL^–1^, adding the phages during the log phase (*t* = 4 h). Phage only samples represent fresh bacteriophage controls, LB media was used as a negative control, and 1% v/v Chemgene was used as a positive control. All other cryopreserved samples used 10 mg·mL^–1^ of each polymer per aliquot. Each growth curve represents three biological replicates and three technical replicates.

To enable screening, all polymers were first screened using a 96-well microplate assay which we have previously used for phage inhibition screening, Figure 2B.^59^ Five distinct phage (K1F-GFP, K1E, K1-5, T7, and T4) were employed. The host for K1F-GFP, K1E, and K1-5 phages was *E. coli* EV36, whereas the host for T7 and T4 phages was *E. coli* K-12 (MG1655 cells). As with the PEG data above, a phage has been successfully cryopreserved if there is a decrease in bacterial growth (judged by a drop in the OD600 measurement), seen at approximately 4 h postinoculation. The growth curves are shown in Figure 3, including a total of 25 polymers, against the five phage, so 125 screening experiments represent, to our knowledge, the largest screen of polymers for this application area.

The screening data confirm that for the neutral (uncharged) polymers (PNIPAM, PHEA, PPEGMA), all molecular weights could protect the phage during cryopreservation, as judged by their bacterial-lytic activity. This is important, as it suggests that the polymer may not interact with the phage itself but provides some other function, as we would not expect all polymers to interact equally. The anionic polymers tested (PAA and PMA) appear to show a reduced cryoprotective function. However, this is not a strictly correct interpretation, as PAA and PMA have been recently shown to inhibit phage infection in bacteria,^59^ hence, PAA/PMA in the mixture might protect the phage against cryo-damage, but also functions to prevent their replication in bacteria. This is explored later in this paper (see Figures 7 and 8).

**Table 1:** Polymer Characterization

polymer code	[M]:[CTA] (−)	*M*_n,SEC_ (g·mol^–1^)	*Đ*^a^ (−)	DP^b^ (−)
PPEGMA 35	25	12600	1.36	35
PPEGMA 40	25	14400	1.10	40
PPEGMA 57	50	20500	1.09	57
PPEGMA 77	50	27700	1.65	77
PPEGMA 102	100	36800	1.10	102
PPEGMA 134	100	48300	2.75	134
PPEGMA 222	300	79800	2.04	222
PPEGMA 382	500	137600	4.15	382
PMA 89	25	7700	1.17	89
PMA 154^c^	50	13300	1.11	154
PMA 155^c^	50	13200	1.19	155
PMA 208	100	18000	1.16	208
PMA 213	100	18400	1.21	213
PMA 278	200	24000	1.14	278
PMA 329	200	28200	1.21	329
PMA 406	500	35000	1.27	406
PAA 73	25	5300	1.18	73
PAA 32	50	2274	1.10	32
PAA 153	100	11000	1.28	153
PAA 187	200	13500	1.26	187
PAA 372	500	26800	1.34	372
PHEA 30^d^	25	3500	1.19	30
PHEA 45	25	5200	1.30	45
PHEA 39^d^	50	4500	1.37	39
PHEA 86	50	9900	1.22	86
PHEA 78	100	8900	1.36	78
PHEA 189^d^	75	21800	1.12	189
PHEA 199	200	22900	1.31	199
PHEA 227	150	26100	1.63	227
PHEA 279	500	32100	1.43	279
PNIPAM 37	25	4100	1.18	37
PNIPAM 35	50	3900	1.22	35
PNIPAM 96	100	10800	1.22	96
PNIPAM 31	200	3500	1.27	31
PNIPAM 119	200	13500	1.52	119
PNIPAM 161	500	18200	1.52	161
PNIPAM 284	500	32100	1.20	284

a Dispersity, *M*_w_/*M*_n_.

b Number average degree of polymerization from SEC data.

c These samples are different batches, which had very similar SEC MW.

d Represents RAFT photopolymerization. The rest are all thermal polymerizations. See Supporting Information for details.

**Figure 4:**
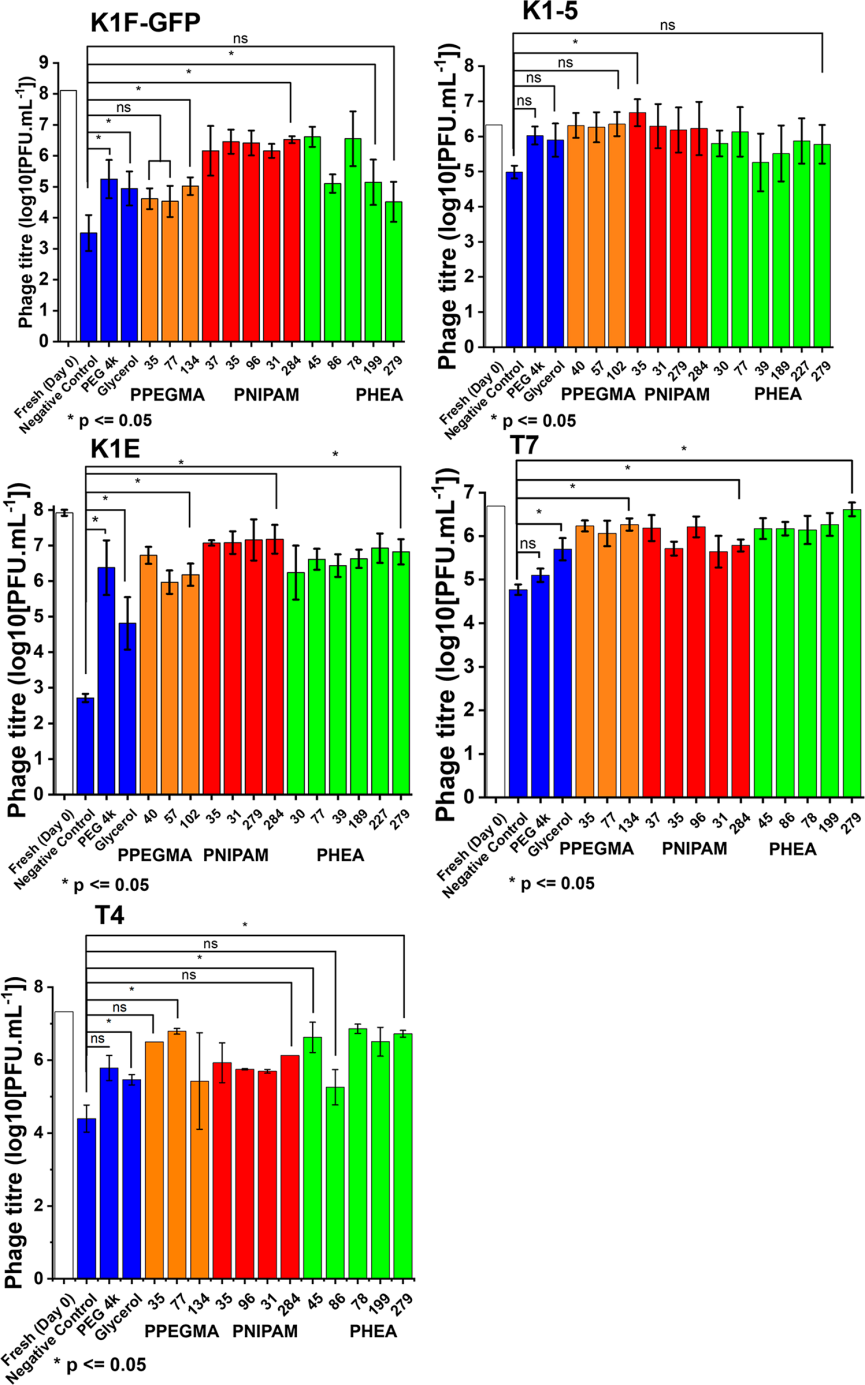
Recovered post-thaw bacteriophage titer after cryopreservation with the polymer library. *E. coli* EV36 was used as host for K1F-GFP, K1-5, and K1E phages; *E. coli* K-12 was used as host for T7 and T4 phages. Phage titer (concentration) comparison across the five bacteriophages. White “Fresh (Day 0)” control represents precryopreservation phage titer. Cryopreserved control, PPEGMA, PNIPAM, and PHEA samples are color coded in blue, orange, red, and green, respectively. Negative control represents no cryoprotectant phage cryopreservation. [PEG 4K]/[Glycerol]/[PPEGMA]/[PNIPAM]/[PHEA] = 10 mg·mL^–1^. A one-way analysis of variance (ANOVA) was performed, using Tukey’s correction, to compare phage recovery of each cryoprotectant sample to the negative control. Any significant difference at a 95% confidence interval was marked by an asterisk (*), whereas nonsignificant difference was marked by ns. Brackets for * marked polymer samples were put at the end of the group, for simplicity, and any nanoseconds were marked, where applicable. Each assay was carried out in biological triplicate and technical replicates.

The above observations suggested all polymers work to some extent in protecting the phage during cryopreservation. To gain a more quantitative understanding, a lower throughput, but quantitative, plaque-forming unit (PFU) assay was undertaken on a smaller set of the polymers. In this assay, thawed phages are added to bacterial hosts on the agar, and the number of plaque-forming units can be counted. In general, all the polymers showed an increase in phage PFU recovery, compared to the negative control (no additive), and in some cases allowed almost 100% PFU recovery (within error). It was not possible to say any polymer was particularly “better” than the others, but they all matched or exceeded the performance of a glycerol control (Figure 4). This is important, as it showed the benefit of macromolecular cryoprotectants. In critically evaluation poly(NIPAM) presents an interesting example: it has a lower critical solution temperature (LCST) in the region of 32 °C.^64,65^ Above the LCST, the polymer can precipitate, which can interfere with assays relying on optical density measurements (such as the screening above). However, this thermoresponsive behavior has been widely used as an alternative way to separate polymer from biologics or provide other additional functions.^66,67^ While not explored here (as it has been widely reported), this again highlights the benefits of using macromolecules as opposed to small molecule/solvent cryoprotectants.

**Figure 5:**
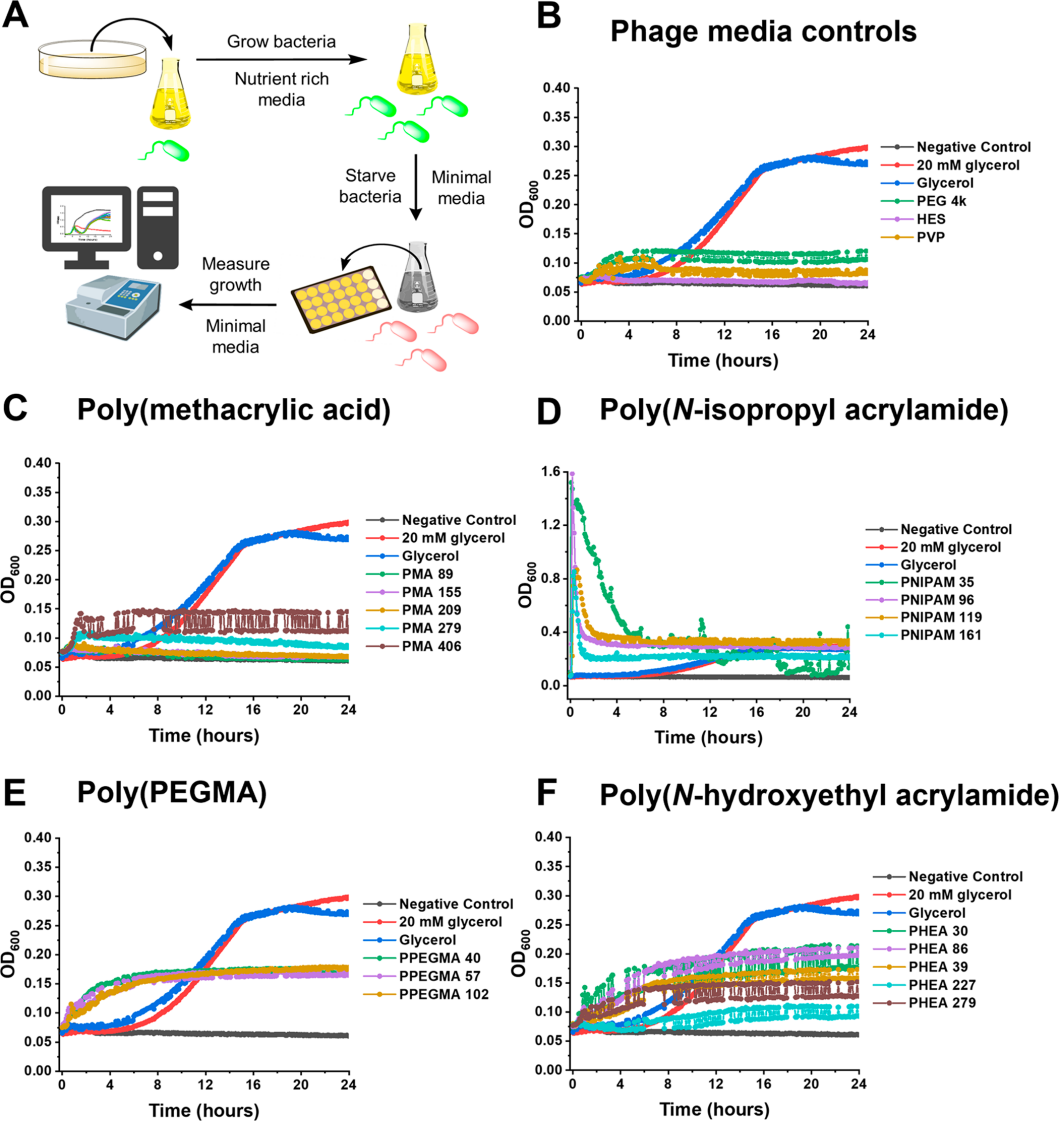
Carbon source testing *E. coli* minimal media growth curves. (A) Schematic of the minimal media growth curve assay. (B) Minimal media growth curves of our phage cryopreservation controls from our previous work.^55^ (C–F) Minimal media growth curves of the polymer library. Starved *E. coli* K-12 (MG1655 cells) was the bacterial strain tested. Negative control represents M9 minimal media only, and 20 mM glycerol was used as positive control. [PEG 4k]/[Glycerol] (not the 20 mM control)/[HES]/[PVP]/[PMA]/[PNIPAM]/[PPEGMA]/[PHEA] = 10 mg·mL^–1^. Growth curves represent one biological replicate and three technical replicates.

The above shows that hydrophilic polymers can be used as a simple replacement for glycerol in phage storage, but it is crucial to state that glycerol itself works very well (and hence is commonly used). However, synthetic polymers can offer some potential advantages. Glycerol is a carbon source for many micro-organisms: they can process and metabolize it to grow.^68^ Hence, a cryopreserved biologic in glycerol if not purified first (to remove glycerol) will be introducing this carbon source which would lead to complications in the study of mechanism of action where starved conditions are often used.^69−71^ Therefore, an experiment was devised to determine if these polymers could act as carbon sources for *E. coli* and *M. smegmatis* as model organisms.^69^ Due to the cell aggregation of *M. smegmatis* complicating optical density (OD) measurements^72^ which led to inconclusive results, *E. coli* was used for the carbon source testing instead. Bacteria were cultured and then starved by several washing steps and replacement of the nutrient-rich LB media with corresponding M9 minimal media. The polymers were then added at 10 mg.mL^–1^, and growth monitored as described above. Figure 5B shows that the addition of glycerol leads to an increase in OD_600_, consistent with bacterial growth (and hence its being a carbon source). In contrast, control polymers of PEG, hydroxyethyl starch (HES) and poly(vinyl pyrrolidinone) (PVP) (Figure 5B) showed only a small increase in OD (due to residual growth capacity of the starved bacteria). Figure 5C–F shows the same curves for the polymer library, and there was no significant growth. PNIPAM in this assay showed some initial aggregation (due to its LCST) which then decreases over time. PPEGMA and PHEA did show small increases which plateaued, but these were consistent with the controls.

**Figure 6:**
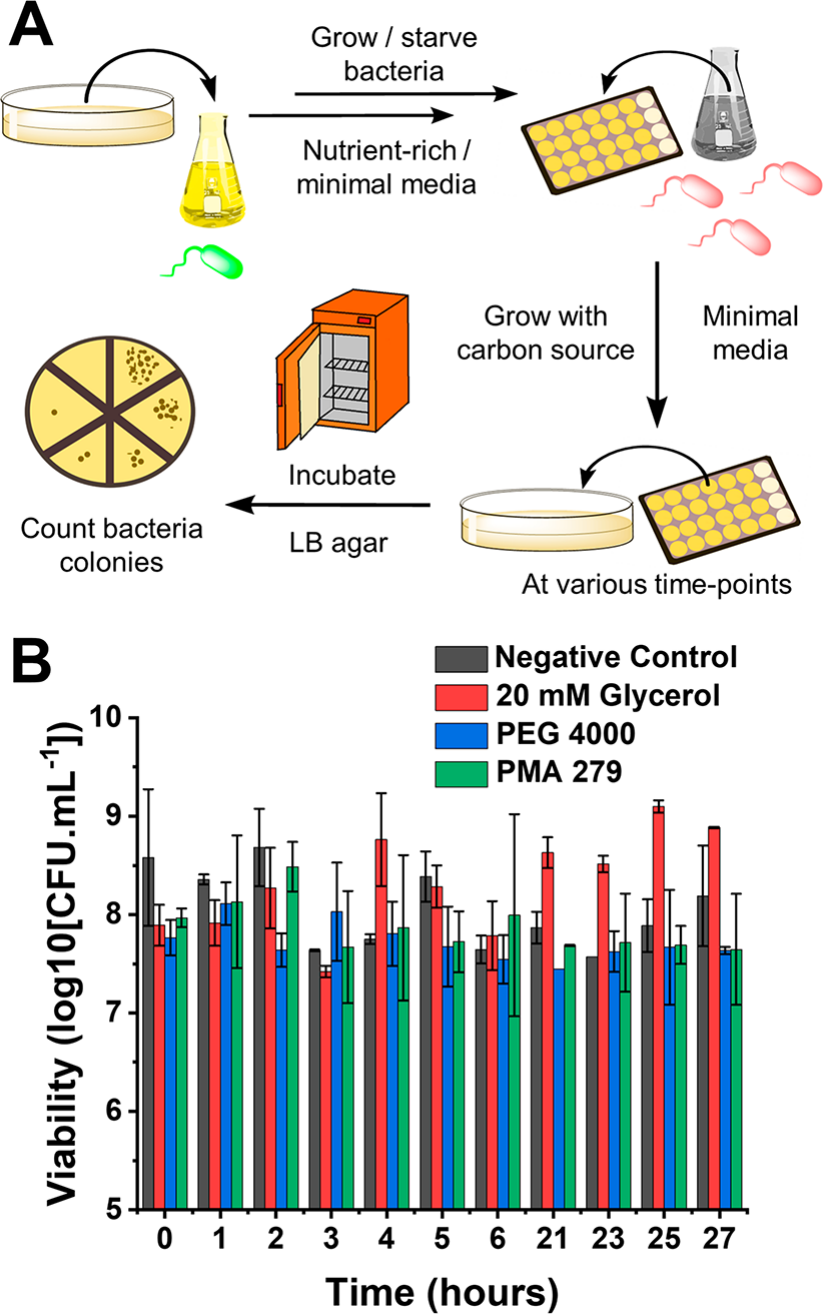
Carbon source testing *E. coli* minimal media time-point bacterial viability assay. (A) Schematic for the minimal media time-point *E. coli* viability assay, highlighting the difference from the growth curve assay. (B) Bacterial growth rate in minimal media over 11 time points, measured as CFU·mL^–1^ (colony forming units). *E. coli* K-12 (MG1655 cells) was used as the model host, with a starting concentration of 1.6 × 10^8^ CFU·mL^–1^ (OD_600_ of 0.2). Aliquots for each time point were extracted from a 96-well plate to mimic the previous assay. Negative control was M9 minimal media, whereas the positive control was 20 mM glycerol. [PEG 4000]/[PMA 279] = 10 mg·mL^–1^.

To further validate the consistency of the small increase shown in PPEGMA, PHEA, and PMA, which do not show an LCST, the bacterial growth rate was monitored by measuring the CFU (colony forming using) over 11 time points, after introducing the polymers to starved cultures. Figure 6 shows that the two polymers (PEG and PMA) added at 10 mg·mL^–1^ did not increase the CFU of *E. coli*, compared to the negative control, whereas the 20 mM glycerol was in fact a carbon source that can be seen after the 21 h time point, when the growth enters an exponential phase (log phase). Therefore, the polymers can be seen to not interfere with the growth nor carbon utilization of the bacteria, unlike glycerol.

**Figure 7:**
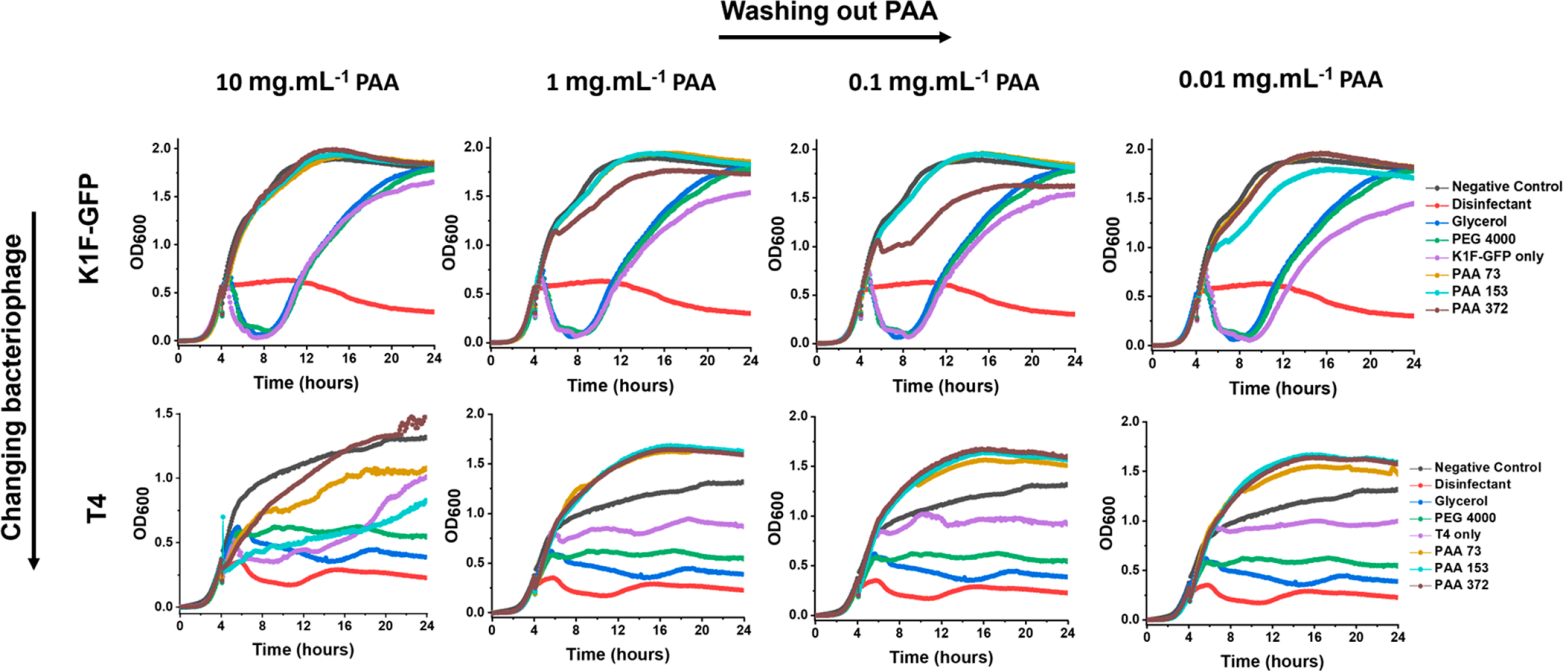
Poly(acrylic acid) washing out postcryopreservation. Dose response growth curves of cryopreserved bacteriophages K1F-GFP and T4 after diluting (washing out) PAA (left to right). Cryopreserved phages in PAA (10 mg·mL^–1^) were 1:10 serially diluted four times to wash out the polymer before addition to the log phage (4 h) host cultures. *E. coli* EV36 was used as the bacteria host for K1F-GFP, whereas *E. coli* K-12 (MG1655 cells) was used as the bacteria host for T4 phages, with a starting concentration of 1 × 10^6^ CFU·mL^–1^. Phage-only controls refer to the diluted nonpolymer containing bacteriophage aliquots that matched the PFU·mL^–1^ (plaque forming units) of each PAA sample within the same condition. LB media was used as negative control, and 1% Chemgene was used as a disinfectant. Growth curves represent biological triplicates and technical duplicates.

**Figure 8:**
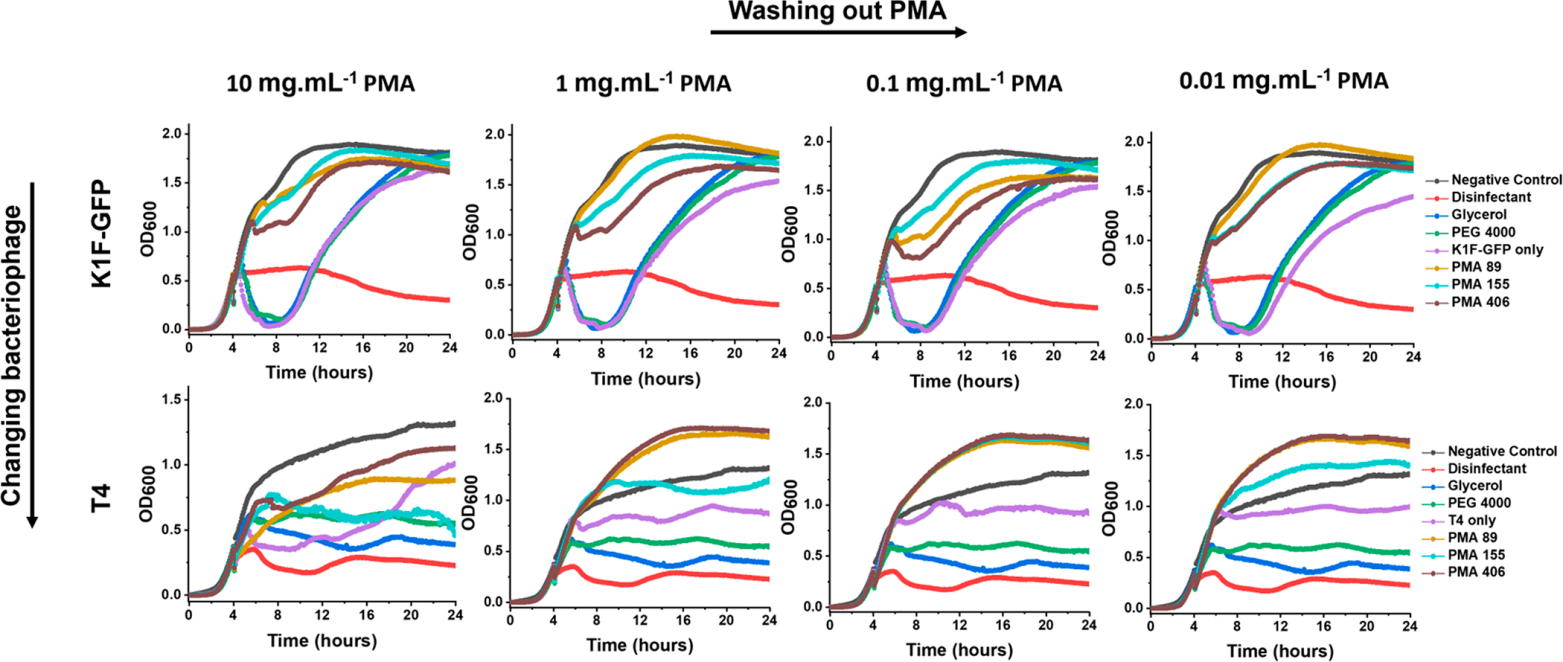
Poly(methacrylic acid) washing out postcryopreservation. Dose response growth curves of cryopreserved bacteriophages K1F-GFP and T4 after dilution (washing out) of PMA (left to right). Cryopreserved phages in PMA (10 mg·mL^–1^) were 1:10 serially diluted four times to wash out the polymer before adding to log phase host cultures. *E. coli* EV36 was used as host for K1F-GFP phages, whereas *E. coli* K-12 (MG1655 cells) was used as host for T4 phages, with starting concentration of 1 × 10^6^ CFU·mL^–1^. Phage-only controls refer to the diluted nonpolymer containing bacteriophage aliquots which matched the PFU·mL^–1^ of each PMA sample within the same condition. LB media was used as negative control and 1% Chemgene as disinfectant. Growth curves represent biological triplicates and technical duplicates.

During the screening above (Figure 3) it was noted that PAA and PMA appeared to not protect the phages from freeze/thaw, as there was no post-thaw bacterial lysis. However, we were concerned this was a false result, as PAA/PMA have been recently reported to be bacteriophage static; they inhibit phage from replicating in bacteria.^59^ This does not rule out their ability to protect from cold stress, and hence an experiment was designed to probe this. In short, phage were cryopreserved with the PAA/PMA libraries (as above), but post-thaw, the polymer was sequentially washed out to reduce the residual concentration during the bacteria-lysis assays from 10 to 0.01 mg·mL^–1^. This was undertaken for the whole bacteriophage library for both polymers, Figures 7 and 8. In most cases, washing of the polymer to 0.01 mg·mL^–1^ did not rescue the function of the phage to infect, with only minimal bacterial lysis. Since phage inhibition and cryoprotection are hard to distinguish postcryopreservation in PAA and PMA, as an additional internal control assay, PAA was diluted to 5 and 2.5 mg·mL^–1^ before incubating with K1F-GFP phage for 24 h, to test its suitability as a cryoprotectant without phage inhibition (Figure S1). A trend was seen between phage recovery and reducing the PAA from 10 to 2.5 mg·mL^–1^, which may remove phage inhibition complications, but the use of PAA/PMA is practically a large barrier.

Considering the above, it seems that phage protection by polymers is a universal approach to enable their cryopreservation and recovery, with all polymers tested capable of mitigating the cold damage compared to the buffer alone. It should be first noted that phages are more robust than other biologics such as proteins or intact cells, which might help explain the apparent ease of increasing recovery. As an additional control mycobacteriophages were also cryopreserved at ultralow freezing temperatures (Figure S1). Mycobacteriophages are usually stored at 4 °C for extended periods.^73,74^ A preliminary cryopreservation of mycobacteriophage ph180 showed slight recovery in phage titer with the addition of PEG, glycerol, hydroxyethyl starch (HES) and poly(vinyl pyrrolidone) (PVP; Figure S2). As the cryoprotective efficacy of PEG and glycerol in the initial screen was much lower for mycobacteriophage, compared to the *E. coli* phages, no further testing with the polymer library was carried out, but it shows that the polymers can perform similarly to glycerol over a range of different phages and are not always better.

A previous detailed study on the use of solvents (DMSO/glycerol) to store a phage (VP3) showed these were not very effective, with only 50% recovery (judged by PFU counts) when stored, and hence, “not everything” can protect them.^75^ In contrast, our approach allowed higher recovery rates for the *E. coli* phage. The exact mechanism of damage to bacteriophage due to cold exposure is difficult to pinpoint due to the challenges of studying it in isolation and hence also the mechanism of protection of the polymers. There is, however, evidence that osmotic pressure is one crucial factor involved: rapid changes in saline concentration, specifically an increase in ionic strength of the solution,^76^ leading to osmotic shock, can inactivate phages and cause DNA extrusion from the phage head, in addition to disjointing of the sheath.^77−79^ Osmotic shock occurs when the phages are cooled below the eutectic temperature of the suspension medium, regardless of rapid- or slow-warming, which suggests the freeze–thaw damage below this temperature is due to the removal of “unbound” water from the suspension to form ice.^80,81^ During freeze-drying of bacteriophage the head coating tends to be damaged, which preserves adsorption and injection abilities, but inhibits colony formation.^82^ Studies on T4 phages have put forward osmotic shock, salt denaturation, and eutectic injury as three potential simultaneously occurring mechanisms leading to freeze–thaw damage in bacteriophages.^81^ During the cryopreservation of the T4 phage, hydrophilic cryoprotectants, including dimethyl sulfoxide (DMSO), poly(vinyl pyrrolidone) (PVP), glycerol, dextran, and sucrose enhanced the recovery, neutralizing the sodium bromide denaturant in the suspension medium.^79^ All additives used were strong hydrogen-bonding compounds.^83^ Hence, a hypothesis for how polymers protect is that they lead to increased viscosity in the unfrozen fraction existing between ice crystals (as ice forms pure phases) and slow the water diffusion rates, mediating osmotic-shock. In general, a polymer solution will have a higher viscosity at equal concentration than a small molecule solution and hence explain why any polymer can benefit phage cryopreservation. Conversely, bacteria are damaged by ice crystals themselves, and so increasing the cryopreservation solution’s viscosity does not have the same beneficial effect seen with phages.^84^ It is again important to note that the polymers are not always superior to glycerol (although they function at lower concentrations) but brings the benefit that the polymers can have additional functionality which might be useful post-thaw, such as handles for purification, to inhibit phage replication selectivity^59^ or as additional functional additives as part of a phage-based therapy.

## Conclusions

Here we explored the question of whether any hydrophilic polymer can protect bacteriophages (phages) during their cryopreservation. Previous reports have shown that PEG had some benefit, but how other polymers function has not been explored. A panel of hydrophilic polymers were first prepared by RAFT polymerization and deployed in a high throughput screening approach against 5 distinct phages: K1F-GFP, K1E, K1-5, T7, and T4. It was observed for each uncharged polymer and phage combination that postcryopreservation recovery (judged by bacterial host lysis) was achieved for every polymer class and all molecular weights. The anionic polymers, poly(acrylic acid), and poly(methacrylic acid), appeared to show no recovery of phage activity (i.e., bacterial growth), but is a false negative, due to their phage-inhibiting properties. Control experiments where the anionic polymers were removed post-thaw showed some rescue of phage activity. Lower throughput, but quantitative, plaque forming unit assays confirmed the results of the screening and validated that the polymers can match or outperform glycerol as a cryoprotectant at 10-fold lower concentration. In some cases, the polymers did outperform glycerol, but a specific benefit was observed, postthaw, that the polymers are no carbon sources for the bacteria to metabolize. Residual glycerol is a carbon source for the bacteria and hence promotes growth. Hence, polymers are passive agents for post-thaw investigation of phage function and may reduce cross-interactions in functional studies. Finally, synthetic polymers can be tuned for easy removal or to bring about additional function, which may help the development of phage-based therapeutics in the future.

## Data Availability

Background data is available in the Supporting Information and at wrap.warwick.ac.uk.

## Supporting Information Available

Additional methods, synthetic information, and underpinning data (PDF)
