# Supplementary material for: Screening of Hydrophilic Polymers Reveals Broad Activity in Protecting Phages during Cryopreservation
Source: Biomacromolecules. 2023 Dec 21;25(1):413–24. doi: 10.1021/acs.biomac.3c01042 (PMC10777348; doi:10.1021/acs.biomac.3c01042)
Supplement: Supplementary file 1 — bm3c01042_si_001.pdf [file bm3c01042_si_001.pdf]

Supporting Information

"Screening of Hydrophilic Polymers Reveals Broad Activity in Protecting Phages During Cryopreservation"

Huba L. Marton,[a] Apoorva Bhatt,[e,f] , Antonia P. Sagona,[c]*, Peter Kilbride,[d] and Matthew I. Gibson[a,b,g,h] *

a) Department of Chemistry, University of Warwick, Coventry, CV4 7AL, United Kingdom

b) Warwick Medical School, University of Warwick, Coventry, CV4 7AL, United Kingdom

c) School of Life Sciences, University of Warwick, Coventry, CV4 7AL, United Kingdom

d) Asymptote, Cytiva, Chivers Way, Cambridge, CB24 9BZ

e) School of Biosciences, University of Birmingham, Birmingham, B15 2TT

f) Institute of Microbiology and Infection, University of Birmingham, Birmingham, B15 2TT

g) Department of Chemistry, University of Manchester, Oxford Road, Manchester, M13 9PL, UK

h) Manchester Institute of Biotechnology, University of Manchester, 131 Princess Street, Manchester, M1 7DN, UK

CORRESPONDING AUTHOR DETAILS

*Fax: +44 247 652 4112. E-mail: matt.gibson@manchester.ac.uk and A.Sagona@warwick.ac.uk

Experimental Section

Materials

All chemicals were used as supplied unless stated otherwise. 1-Dodecanethiol (≥98%), 2-bromo-2-methylpropionic acid (98%), carbon disulfide (anhydrous, ≥99%), tripotassium phosphate (≥98%), hydrochloric acid (reagent grade), dichloromethane (≥99.8%), ethyl acetate (99.8%), *N,N*-dimethylformamide (99%, DMF), tetrahydrofuran (≥99.9%, THF), diethyl ether (≥ 99.7%), n-hexane (reagent grade), petroleum ether (boiling point 40-60 °C) silica gel, 4,4'-azidobis(4-cyanovaleric acid) (≥75%, ACVA), poly(ethylene glycol) methacrylate (M_n 360, PEGMA), methacrylic acid (99%, MA), acrylic acid (99%, AA), *N*-hydroxyethyl acrylamide (97%, HEA), *N*-isopropyl acrylamide (97%, NIPAM), 4-cyano-4-(phenylcarbonothioylthio)pentanoic acid, 2-(dodecylthiocarbonothioylthio)-2-methylpropionic acid 3-azido-1-propanol ester (98%), 2-cyano-2-propyl dodecyl trithiocarbonate (97%), 4-cyano-4-[(dodecylsulfanylthiocarbonyl)sulfanyl]pentanoic acid (97%), chloroform-d_1 (99.8%), methanol-d_4 (≥99.8%), deuterium oxide (99.9%), agarose, lysogeny broth (LB), poly(ethylene glycol) PEG (Mn 4,000), polyvinylpyrrolidone (PVP) (Mn 40,000), polyethylene glycol sorbitan monooleate (Tween80), sodium phosphate dibasic heptahydrate (≥99%), and potassium phosphate monobasic (≥99%) were purchased from Sigma-Aldrich (Merck). Calcium chloride (≥99%), caesium chloride (≥98%), magnesium sulfate heptahydrate (≥98%), sodium chloride (≥99%), ammonium chloride (≥99%) and PEG (Mn 8,000) were purchased from Fisher Scientific. (Glycerol (≥99%) was purchased from Scientific Laboratory Supplies (SLS). Hydroxyethyl starch (HES) was purchased from Carbosynth. Phosphate buffered solution (PBS) (8 $g.L^{-1}$ NaCl, 0.2 $g.L^{-1}$ KCl, 1.15 $g.L^{-1}$ Na_2HPO_4, 0.2 $g.L^{-1}$ KH_2PO_4) and Tris-HCl (24.2 $g.L^{-1}$ Tris, 80 $g.L^{-1}$ NaCl) was provided by media preparation facility in the School of Life Sciences at the University of Warwick. Suspension medium (SM-I) (1 M NaCl, 8 mM $MgSO_4.7H_2O$, 22.5 mM Tris-HCl pH 7.5), SM-

II (100 mM NaCl, 8 mM $MgSO_4.7H_2O$, 22.5 mM Tris-HCl pH 7.5), MP buffer (150 mM NaCl, 10 mM $MgSO_4$, 50 mM Tris-HCl, pH 7.5 and 2 mM $CaCl_2$) and M9 minimal media (12.8 g.L^{-1} $Na_2HPO_4.7H_2O$, 3 g.L^{-1} KH_2PO_4, 0.5 g.L^{-1} NaCl, 0.1 g.L^{-1} NH_4Cl, 2 mM $MgSO_4$ and 0.1 mM $CaCl_2$) were prepared in house.

Physical and Analytical Methods

NMR spectroscopy.

Proton (^{1}H-NMR) nuclear magnetic resonance spectra were recorded at 300 MHz or 400 MHz on a Bruker DPX-300 or DPX-400 spectrometer respectively, with chloroform-d ($CDCl_3$), dimethyl sulfoxide-d6 ($(CD_3)_2SO$), deuterium oxide (D_2O) and methanol-d4 (CD_3OD) as the solvents. Chemical shifts of protons are reported as δ in parts per million (ppm) and are relative to tetramethylsilane (TMS) at δ = 0 ppm when using $CDCl_3$ or solvent residual peak (CH_3OH, δ = 3.31 ppm) for CD_3OD and ($(CH_3)_2SO$, δ = 2.54 ppm) for $(CD_3)_2SO$, (H_2O, δ = 4.78 ppm).

Fourier Transformed-Infrared (FT-IR) spectroscopy.

Fourier-Transform-Infrared (FT-IR) spectroscopy measurement of each synthesized compound was carried out in the range of 650 to 4000 cm^{-1} using a Cary 630 FT-IR spectrometer (Agilent).

Size exclusion chromatography (SEC) in DMF.

For each polymer, size exclusion chromatography (SEC) analysis was performed on an Agilent Infinity II MDS instrument equipped with differential refractive index (DRI), viscometry (VS), dual angle light scatter (LS) and variable wavelength UV detectors. The system was equipped with 2 × PLgel Mixed D columns (300 × 7.5 mm) and a PLgel 5 µm guard column. The mobile phase used was DMF (HPLC grade) containing 5 mM NH_4BF_4 at 50 °C at a flow rate of 1.0 mL.min^{-1}. Poly(methyl methacrylate) (PMMA) standards (Agilent EasyVials) were used for calibration between 955,000 – 550 g.mol^{-1}. Analyte samples were filtered through a nylon membrane with 0.22 μm pore size before injection. Number average molecular weights (M_n),

average molecular weights (M_w) and dispersities ($Đ_M$ = M_w/M_n) were determined by conventional calibration and universal calibration using Agilent GPC/SEC software.

Synthesis of *2-(dodecylthiocarbonothioylthio)-2-methyl propionic acid (DMP)*

The synthesis of DMP was carried out according to a previous procedure[1]. 1-dodecane thiol (2.00 g, 9.88 mmol) was slowly added to stirring K_3PO_4 (2.10 g, 9.89 mmol) in acetone (30 mL) at RTP (room temperature and pressure), leaving the new mixture stirring for 25 minutes until white suspension formed. After adding carbon disulphide (2.05 g, 26.93 mmol) the mixture was further stirred for 10 minutes, to form a yellow solution. 2-bromo-2-methyl-propionic acid (1.5 g, 8.98 mmol) was then added, and solution left stirring for 16 hours. Solvent was removed under vacuum. Crude product was dissolved in 1M HCl (100 mL) and extracted with DCM (2 × 100 mL). Organic layer was washed with water (200 mL) and brine (200 mL), dried with $MgSO_4$ and filtered under gravity. Solvent from the filtrate was removed under vacuum. The crude product was purified through a silica column (40-60 PET : DCM : glacial acetic acid 75:24:1) and recrystallized in n-hexane to give a yellow solid (32%). ^{1}H NMR (300 MHz, $CDCl_3$) δ = 3.30 (2H,t, $SCH_2\mathbf{CH_2}$), 1.75 (6H, s, $C\mathbf{(CH_3)_2}$), 1.69 (2H, qn, $S\mathbf{CH_2}$), 1.46 - 1.22 (18H, m,$\mathbf{(CH_2)}_9CH_3$), 0.93 - 0.87 (3H, m, $\mathbf{CH_3}$). m/z calculated as 364.16; found for ESI $[M+H]^+$ 365.1 and $[M+Na]^+$ 387.1. FTIR (cm^{-1}) – 2956, 2917 & 2848 (methyl and methylene), 1702 (ester C=O), 1459, 1437 & 1413 (methyl and methylene), 1280 ($C\mathbf{(CH_3)_2}$), 1064 (S-C(S)-S).

Synthesis of Poly(poly(ethylene glycol) methacrylate)) (PPEGMA)

The following polymers: poly(poly(ethylene glycol) methacrylate; poly(methacrylic acid); poly(acrylic acid); poly(*N*-hydroxyethyl acrylamide and poly(*N*-isopropyl acrylamide) were synthesized using previously used procedures[1].

As a representative, poly(ethylene glycol) methacrylate (M_n 360) (1.29 g, 3.58 mmol, 50 eq), 4-cyano-4-(phenylcarbonothioylthio) pentanoic acid (0.02 g, 0.07 mmol, 1 eq), 4,4'-

azidobis(4-cyanovaleric acid) (0.004 g, 0.014 mmol, 0.2 eq), DMF (6.6 mL) were added and mixed in a glass vial. After sealing with a Suba seal, solution bubbled with nitrogen (purged) for 20 minutes, and reaction left stirring at 60 ºC overnight, after taking a small sample to determine conversion. Next day, the reaction was quenched by submerging the glass vial in liquid nitrogen and exposing solution to air. Crude product was precipitated from diethyl ether (2 × 50 mL) and dried under vacuum (using a Schlenk line / vacuum gas manifold). The resulting polymer was analysed by ^{1}H NMR, and SEC. Representative characterization data for $PPEGMA_{50}$: ^{1}H NMR (400 MHz, DMSO-d_6): δ: 7.87 – 7.43 (5H, m, Ar), 4.61 – 4.47 (3H, s, CNC**(CH$_3$)**CH2CH2), 4.06 – 3.96 (4H, m, CNC(CH$_3$)**CH2CH2**), 3.67 – 3.46 (4H, m, COO**CH2CH2**OCH$_3$), 3.38 – 3.28 (3H, s, COOCH2CH2O**CH$_3$**) 2.10 – 1.52 (2H, m, SCSC(CH$_3$)**CH$_2$**), 1.02 – 0.62 (3H, m, SCSC(**CH$_3$**)CH$_2$). M_n^{SEC} (DMF) = 27700 g.mol^{-1}, $Đ_M$ = 1.65.

Synthesis of Poly(methacrylic acid) (PMA)

As a representative example, methacrylic acid (0.31 g, 3.58 mmol, 100 eq), 4-cyano-4-(phenylcarbonothioylthio) pentanoic acid (0.01 g, 0.036 mmol, 1 eq), 4,4’-azidobis(4-cyanovaleric acid) (0.002 g, 0.0072 mmol, 0.2 eq), methanol (1.6 mL) as solvent were added to a glass reaction vial. A stirrer bar was added, vial sealed and solution purged with nitrogen for 20 minutes. Small sample taken to determine conversion (NMR) and reaction was left stirring at 60 ºC overnight. Next day, the reaction was quenched in liquid nitrogen and exposure to air. Crude polymer was precipitated from diethyl ether and dried under vacuum. Final product was analysed by ^{1}H NMR, and SEC. Representative characterization data for PMA_{100}: ^{1}H NMR (400 MHz, CD_3OD): δ 7.93 – 7.34 (5H, m, Ar), 3.78 – 3.58 (3H, m, C**(CH$_3$)**CNCH$_2$CH$_2$), 2.29 – 1.74 (2H, m, SCSC(CH$_3$)(COOH)**CH$_2$**), 1.72 – 1.47 (2H, m, COOH**CH$_2$**CH$_2$)), 1.46 – 1.29 (2H, m, COOHCH$_2$**CH$_2$**), 1.15 – 1.00 (3H, m, SCSC(**CH$_3$)**(COOH)CH$_2$). M_n^{SEC} (DMF) = 18400 g.mol^{-1}, $Đ_M$ = 1.21.

Synthesis of Poly(acrylic acid) (PAA)

As a representative example, acrylic acid (1.98 g, 27.46 mmol, 100 eq), previously synthesized DMP (0.1 g, 0.27 mmol, 1 eq), 4,4'-azidobis(4-cyanovaleric acid) (ACVA) (0.015 g, 0.055 mmol, 0.2 eq) and methanol (10.5 mL) were added to a glass vial. A stirrer bar was added, the vial sealed, solution degassed with nitrogen for 20 minutes, a small sample taken to determine conversion by NMR and reaction left stirring at 60 ºC overnight. Next day, the reaction was quenched by submerging the glass vial in liquid nitrogen and exposing solution to air. Crude polymer was precipitated from diethyl ether and dried under vacuum. The resulting polymer product was analysed by ^{1}H NMR, and SEC. Representative characterization data for PAA_{100}: ^{1}H NMR (400 MHz, CD_3OD): δ 3.45 – 3.38 (4H, m, C(S)S**$(CH_2)_2$**CH_2CH_2), 2.78 – 2.66 (6H, m, COOHC**$(CH_3)_2$**), 2.64 – 2.25 (1H, m, S**CH**(COOH)CH_2) , 2.22 – 1.48 (2H, m, SCH(COOH)**CH_2**) 1.46 – 1.28 (18H, m, CH_3**$(CH_2)_9$**CH_2), 0.96 – 0.89 (3H, t, **CH_3**$(CH_2)_{11}$SCS). M_n^{SEC} (DMF) = 11000 g.mol^{-1}, $Đ_M$ = 1.28.

Alternative chain transfer agent used was 2-Cyano-2-propyl dodecyl trithiocarbonate.

Synthesis of Poly(N-hydroxyethyl acrylamide) (PHEA)

As a representative example, *N*-hydroxyethyl acrylamide (1.00 g, 8.68 mmol, 100 eq), 2-cyano-2-propyl dodecyl trithiocarbonate (0.03 g, 0.087 mmol, 1 eq), ACVA (0.0049 g, 0.018 mmol, 0.2 eq) and methanol (5.2 mL) were added to a glass vial. The vial sealed, solution purged with nitrogen for 20 minutes, a small sample taken to determine conversion and reaction left stirring at 60 ºC overnight. Next day, the reaction was quenched by submerging the glass vial in liquid nitrogen and exposing solution to air. Crude polymer product was precipitated from diethyl ether and dried under vacuum. Final product was analysed by ^{1}H NMR, and SEC. Representative characterization data for $PHEA_{100}$: ^{1}H NMR (400 MHz, CD_3OD): δ 8.16 – 7.94 (1H, m, CO**NH**CH_2CH_2OH), 3.81 – 3.52 (2H, m, CONHCH_2**CH_2**OH), 3.41 – 3.06 (2H, t, CONH**CH_2**CH_2OH), 2.4 – 1.77 (1H, m, S**CH**(CONHCH_2CH_2OH)CH_2), 1.77 – 1.30 (2H, m,

SCH(CONHCH$_2$CH$_2$OH)**CH$_2$**), 1.30 – 1.20 (6H, s, CNC**(CH$_3$)$_2$**), 0.79 – 0.69 (3H, t, **CH$_3$**(CH$_2$)$_{11}$S) (CH$_2$ protons from the 12 carbon end of the RAFT agent were overlapped by two backbone CH$_2$). M_n^{SEC} (DMF) = 8900 g.mol^{-1}, $Đ_M$ = 1.36.

Alternative chain-transfer agent used was 4-cyano-4-[(dodecylsulfanylthiocarbonyl)sulfanyl] pentanoic acid.

Photo-synthesis of Poly(N-hydroxyethyl acrylamide) (PHEA)

Additionally, some PHEA polymers were photo-polymerized using a previously utilized procedure[1] (marked with asterisk (*) in Table 1). Pentafluorophenyl-2-dodecylthiocarbonothioylthio)-2-methylpropanoate (PFP-DMP) was kindly provided by Dr Alexander Baker. *N*-hydroxyethyl acrylamide (1.08 g, 9.42 mmol, 50 eq), PFP-DMP (0.1 g, 0.19 mmol, 1 eq), and methanol (5.9 mL) were mixed in a glass vial. The vial was sealed, mixture degassed with nitrogen for 20 minutes, a small sample taken to determine conversion and reaction left stirring at RTP under blue light (460 nm) for 5 hours. Afterwards, the reaction was quenched by submerging the glass vial in liquid nitrogen and exposing solution to air. Crude product was precipitated from diethyl ether (2 × 50 mL) and dried under vacuum. The final polymer product was analysed by ^{1}H NMR, and SEC. Representative characterization data for PHEA$_{50}$: ^{1}H NMR (400 MHz, CD$_3$OD): δ 8.28 – 7.90 (1H, m, CO**NH**CH$_2$CH$_2$OH), 3.95 – 3.45 (2H, t, CONHCH$_2$**CH$_2$**OH), 3.44 – 3.22 (2H, t, CONH**CH$_2$**CH$_2$OH), 2.29 – 1.92 (1H, m, S**CH**(CONHCH$_2$CH$_2$OH)CH$_2$), 1.90 – 1.45 (2H, m, SCH(CONHCH$_2$CH$_2$OH)**CH$_2$**), 1.34 – 1.26 (14H, t, CH$_3$CH$_2$**(CH$_2$)$_7$**), 0.99 – 0.85 (3H, t, **CH$_3$**(CH$_2$)$_2$S) (unaccounted CH$_2$ protons from the 12 carbon end of the RAFT agent, and the 2 × CH$_3$ near PFP end of RAFT agent were overlapped by backbone proton peaks). M_n^{SEC} (DMF) = 4500 g.mol^{-1}, $Đ_M$ = 1.37.

Alternative chain-transfer agent used was 4-cyano-4-[(dodecylsulfanylthiocarbonyl)sulfanyl] pentanoic acid.

Synthesis of Poly(N-isopropyl acrylamide) (PNIPAM)

As a representative example, *N*-isopropyl acrylamide (1.55 g, 13.73 mmol, 100 eq), DMP (0.05 g, 0.14 mmol, 1 eq), 4,4'-azidobis(4-cyanovaleric acid) (ACVA) (0.0077 g, 0.0028 mmol, 0.2 eq), THF (8.1 mL) were added to a glass vial. After adding a stirrer bar, the vial sealed with a Suba seal, solution purged with nitrogen for 20 minutes, a small sample taken to determine conversion and reaction left stirring at 60 °C overnight. Next day, the reaction was quenched by submerging the glass vial in liquid nitrogen and exposing solution to air. Crude polymer was precipitated from diethyl ether (twice) and dried under vacuum. Final product was analysed by ^{1}H NMR, and SEC. Representative characterization data for $PNIPAM_{100}$: ^{1}H NMR (400 MHz, CD_3OD): δ 8.21 – 7.82 (1H, m, CO**NH**$CH(CH_3)_2$), 4.13 – 3.85 (1H, m, CONH**CH**$(CH_3)_2$), 3.80 – 3.70 (2H, t, S**CH_2**$(CH)_{10}CH_3$), 2.33 – 1.93 (1H, m, S**CH**$(CONHCH(CH_3)_2)CH_2$), 1.92 – 1.86 (6H, t, C**$(CH_3)_2$**COOH), 1.83 – 1.38 (2H, m, $SCH(CONHCH(CH_3)_2)$**CH_2**), 1.38 – 1.28 (10H, t, CH_3**$(CH_2)_5$**CH_2), 1.27 – 1.06 (6H, s, SCH(CONHCH**$(CH_3)_2$**$)CH_2$), 0.95 – 0.89 (3H, t, **CH_3**$(CH_2)_{11}S$) (unaccounted CH_2 of the 12 carbon end of the RAFT agents were overlapped by backbone CH_2 protons). M_n^{SEC} (DMF) = 10800 g.mol^{-1}, $Đ_M$ = 1.22.

Alternative chain transfer agent used was 2-Cyano-2-propyl dodecyl trithiocarbonate.

Biological methods

Viral enrichment – propagation of ph180 mycobacteriophages.

Mycobacterium Smegmatis MC^2 155 host and ph180 mycobacteriophage was kindly provided by Dr Apoorva Bhatt from the Institute of Microbiology and Infection (IMI), School of Biosciences, University of Birmingham as part of a collaboration. The propagation and purification steps described below were performed by Albel Singh from Bhatt lab group, at the same institute.

To propagate mycobacteriophage isolates, fresh *Mycobacterium Smegmatis* MC^2 155 host culture was allowed to grow for 3 days with 0.05% v/v of Tween80 . After back dilution, the culture was allowed to outgrow to OD_{600} of 1.0, followed by centrifugation at 2140 g for 10 min, and two MP buffer washes to remove any Tween80 left in the media. Using 100 µL of *M. Smegmatis* host cells, 150 µL of serially diluted temperature sensitive mycobacteriophage ph180 (between dilutions -2 to -8) in MP buffer was mixed for each dilution, followed by plating on lysogeny broth agar (LBA, 1.5% agar) plates using 4 mL of LBA overlay (or soft agar, 0.6% agar), rotating the plates to ensure even spread of the molten overlay. Once soft / top agar solidified, the plates were incubated at 30 °C for 3 days to let the phage plaques fully develop. Plates showing a 'lacy pattern' were selected, where the plaques were distinguishable from each other, but white streaks indicated the growth of the *M. Smegmatis* host. To each selected plate, 3 mL of MP buffer was added and each plate left on a plate rocker overnight. The next day, from each selected plate, the phage containing MP buffer was collected a pooled into a sterile conical flask. Mycobacteriophage lysate was passed through a 5 µm filter followed by a 0.2 µm filter for complete sterilization, and removal of any leftover host. Purified mycobacteriophages were stored at 4 °C.

Plaque assay – quantification of mycobacteriophages.

Mycobacteriophage titres of ph180 phages were determined via the previously mentioned soft agar plaque assay, similarly using 0.7% agar top LBA. *Mycobacteria smegmatis* MC^2 155 host was previously grown with 0.05% v/v Tween80 in LB media at 37 °C in a shaking incubator at 150 RPM over 2-3 days. The culture was back diluted to OD_{600} of 1.0 (~ 1 x 10^9 $CFU.mL^{-1}$ (colony forming units)), to dilute out any Tween80. From the previously cryopreserved aliquots, 100 µL of serially diluted phage were mixed with 140 µL of mycobacteria host cell lawn (~ 1 x 10^9 $CFU.mL^{-1}$) at room temperature, before the addition of 3 mL liquid top agar (0.7% agar containing 1 mM of $CaCl_2$) and pouring over a solid 1.5% agar LBA plate. After 4

day incubation at 30 °C, the plaques were counted and quantified as PFU.mL^{-1} (plaque forming units) taking into account the serial dilution from frozen aliquots. The assays were carried out in biological triplicate, using duplicates for each repeat (n=6).

Supplementary Data

Pre-cryopreservation phage incubation with poly(acrylic acid).

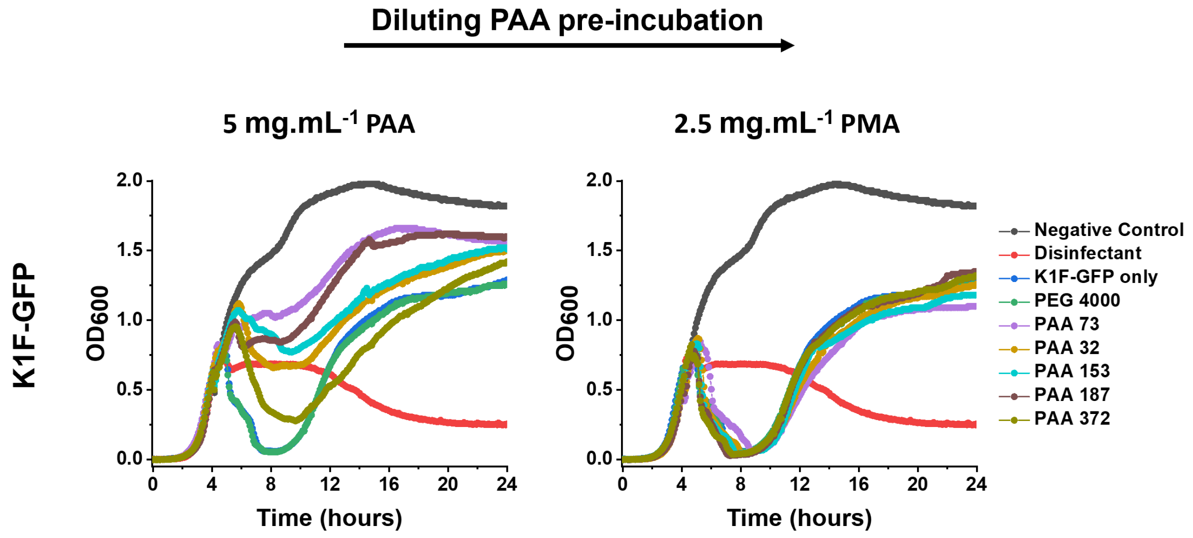

Figure S1. Poly(acrylic acid) pre-cryopreservation dilution. Dose response growth curves of bacteriophage K1F-GFP after 24 hour incubation in diluted PAA. *E. coli* EV36 was used as host for with starting concentration of 1×10^6 CFU.mL^{-1} . K1F-GFP only control refers SM-II buffer incubated aliquots, LB media was used as negative, and 1% Chemgene as disinfectant. [PEG] = 10 mg.mL^{-1}. The dose response growth curves represent one biological and technical triplicates.

Mycobacteriophage Cryopreservation.

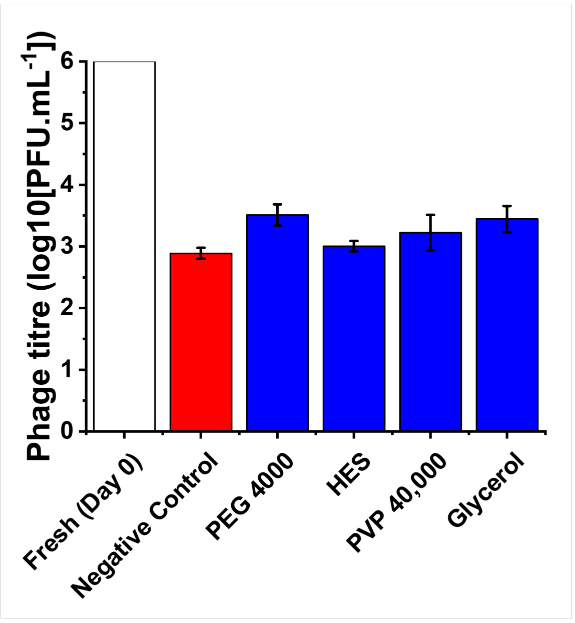

Figure S2. Recovered post-thaw mycobacteriophage titre. *M. smegmatis* MC^2 155 was used as host for ph180 mycobacteriophage. Fresh (Day 0) (white) control represents pre-cryopreservation phage titre. Negative control (red) represents no cryoprotectant phage cryopreservation. [PEG 4000] / [HES] / [PVP 40,000] / [Glycerol] = 10 mg.mL^{-1}. Assays represent biological triplicates and technical triplicates.

Post-cryopreservation polymer washing: poly(acrylic acid) and poly(methacrylic acid)

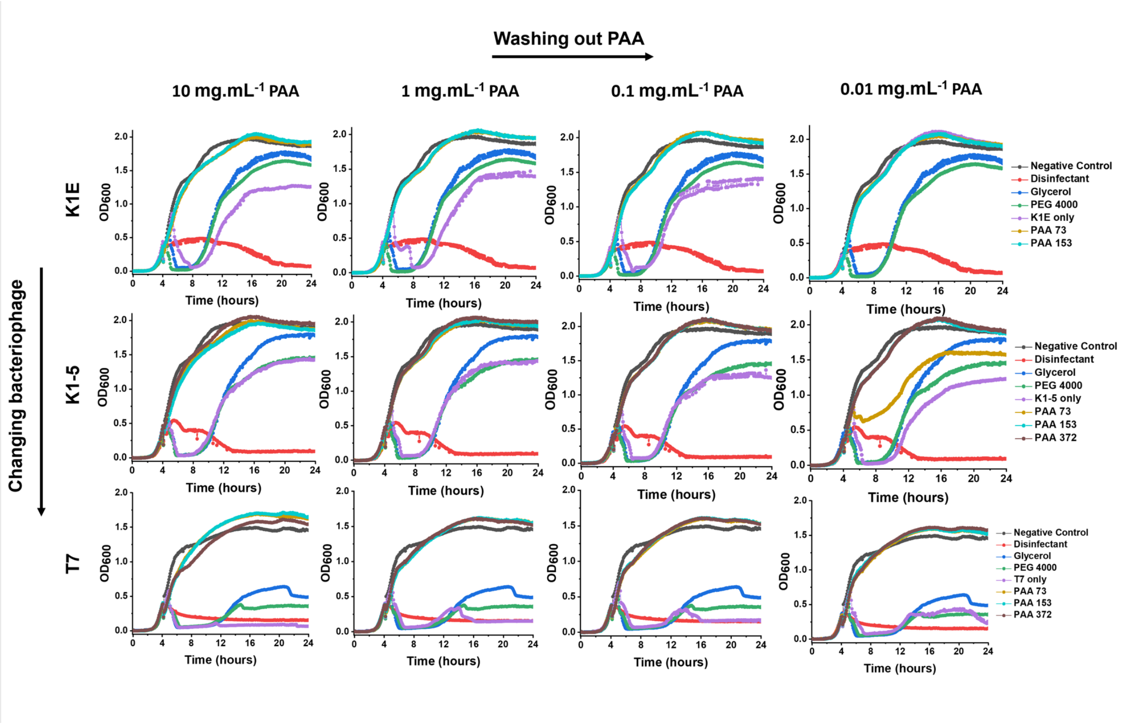

Figure S3. Poly(acrylic acid) washing out post-cryopreservation. Dose response growth curves of cryopreserved bacteriophages K1E, K1-5 and T7 after diluting (washing out) the PAA (left to right). Cryopreserved phages in PAA (10 mg.mL^{-1}) were 1:10 serially diluted four times to wash out the polymer before addition to log phage (4 h) host cultures. *E. coli* EV36 was used as the bacteria host for K1E and K1-5 phages, whereas *E. coli* K-12 (MG1655 cells) was used as bacteria host for T7 phages, with starting concentration of 1×10^6 CFU.mL^{-1} . Phage only controls refer to the diluted non-polymer containing bacteriophage aliquots which matched the PFU.mL^{-1} of each PAA sample within the same tested condition. LB media was used as negative control and 1% Chemgene as disinfectant. Growth curves represent one biological replicate and technical duplicates.

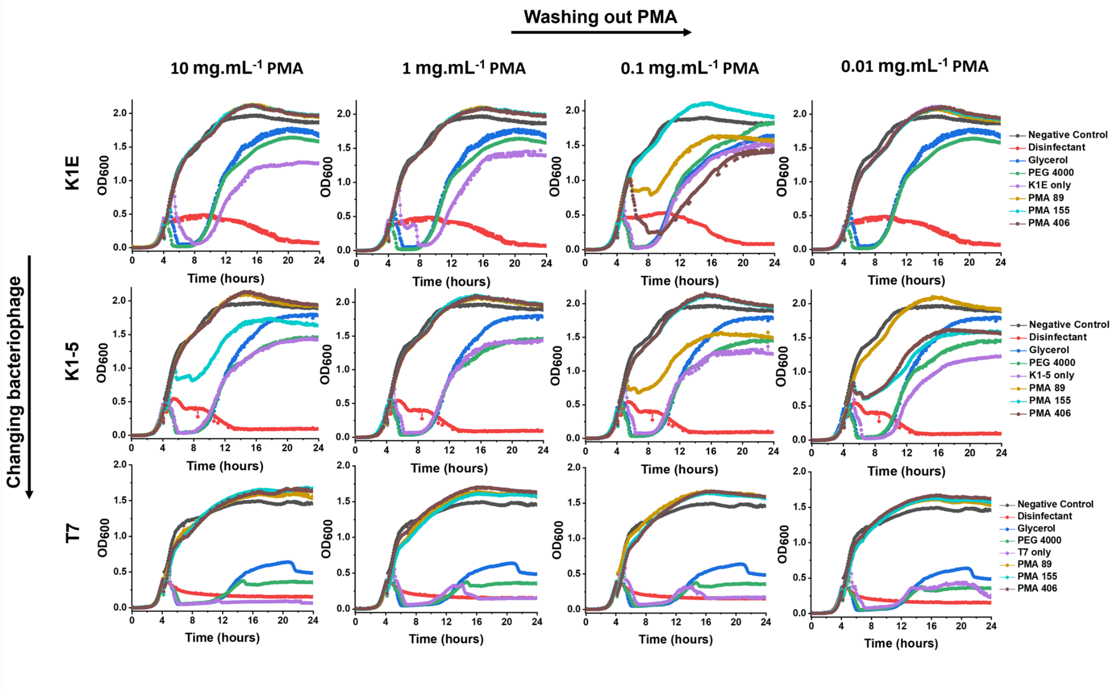

Figure S4. Poly(methacrylic acid) washing out post-cryopreservation. Dose response growth curves of cryopreserved bacteriophages K1E, K1-5 and T7 after diluting (washing out) the PMA (left to right). Cryopreserved phages in PMA (10 mg.mL^{-1}) were 1:10 serially diluted four times to wash out the polymer before addition to log phage (4 h) host cultures. *E. coli* EV36 was used as the bacteria host for K1E and K1-5 phages, whereas *E. coli* K-12 (MG1655 cells) was used as bacteria host for T7 phages, with starting concentration of 1×10^6 CFU.mL^{-1}. Phage only controls refer to the diluted non-polymer containing bacteriophage aliquots which matched the PFU.mL^{-1} of each PMA sample within the same tested condition. LB media was used as negative control and 1% Chemgene as disinfectant. Growth curves represent one biological replicate and technical duplicates.

References

(1) Marton, H. L.; Kilbride, P.; Ahmad, A.; Sagona, A. P.; Gibson, M. I. Anionic Synthetic Polymers Prevent Bacteriophage Infection. *J. Am. Chem. Soc.* **2023**, *145* (16), 8794–8799.
